# LARGE Expression Augments the Glycosylation of Glycoproteins in Addition to α-Dystroglycan Conferring Laminin Binding

**DOI:** 10.1371/journal.pone.0019080

**Published:** 2011-04-20

**Authors:** Zhen Zhang, Peng Zhang, Huaiyu Hu

**Affiliations:** Department of Neuroscience and Physiology, Upstate Medical University, Syracuse, New York, United States of America; Universidade de São Paulo, Brazil

## Abstract

Mutations in genes encoding glycosyltransferases (and presumed glycosyltransferases) that affect glycosylation and extracellular matrix binding activity of α-dystroglycan (α-DG) cause congenital muscular dystrophies (CMDs) with central nervous system manifestations. Among the identified genes, *LARGE* is of particular interest because its overexpression rescues glycosylation defects of α-DG in mutations of not only *LARGE* but also other CMD-causing genes and restores laminin binding activity of α-DG. It is not known whether LARGE protein glycosylates other proteins in addition to α-DG. In this study, we overexpressed LARGE in DG-deficient cells and analyzed glycosylated proteins by Western blot analysis. Surprisingly, overexpression of LARGE in α-DG-deficient cells led to glycosylation dependent IIH6C4 and VIA4-1 immunoreactivity, despite the prevailing view that these antibodies only recognize glycosylated α-DG. Furthermore, the hyperglycosylated proteins in LARGE-overexpressing cells demonstrated the functional capacity to bind the extracellular matrix molecule laminin and promote laminin assembly at the cell surface, an effect that was blocked by IIH6C4 antibodies. These results indicate that overexpression of LARGE catalyzes the glycosylation of at least one other glycoprotein in addition to α-DG, and that this glycosylation(s) promotes laminin binding activity.

## Introduction

Congenital muscular dystrophies (CMDs) with central nervous system and eye malformations such as Walker-Warburg Syndrome (WWS), Muscle-eye-brain disease (MEB), Fukuyama congenital muscular dystrophy (FCMD), and Congenital muscular dystrophy type 1D (MDC1D) can be caused by mutations in genes encoding glycosyltransferases (or putative glycosyltransferases). Some of these genes, which include *POMT1* [1], [2]; *POMT2* [3]; *POMGnT1* [4]; *LARGE* [5]; *FKTN* [6], [7]; and *FKRP* [8], [9], are involved in the synthesis of O-linked mannosyl glycans, such as, Siaα2,3Galβ1,4GlcNAcβ1,2Man-Ser/Thr and Galβ1,4(Fucα1,3)GlcNAcβ1,2Man-Ser/Thr [10]–[12]. Protein O-mannosyltransferase 1 and 2 (POMT1 and 2) form a mutually indispensible enzyme complex and catalyze the first step in the O-mannosyl glycosylation pathway, transferring mannose to serine or threonine residues [13], [14]. Protein O-mannose N-acetylglucosaminyltransferase 1 (POMGnT1) transfers N-acetylglucosamine to O-linked mannose forming a β1,2 linkage [4], [15]. Enzymes that catalyze further extension of O-mannosyl glycans have not been identified. In particular, the substrates of fukutin (encoded by *FKTN*), fukutin-related protein (encoded by *FKRP*), and like-acetylglucosaminyltransferase (LARGE) are not yet fully elucidated. Studies indicate that LARGE modifies O-linked mannosyl glycans, complex N-, and mucin O-glycans [16], [17], and more recent data indicate that LARGE is involved in extension of an unidentified phosphoryl glycosylation branch on O-linked mannose [18].

O-linked mannosyl glycans account for 1/3 of O-linked glycans in the brain [19]–[22]. The only known target of O-mannosyl glycosylation is α-dystroglycan (α-DG), a widely expressed cell surface glycoprotein that binds to the transmembrane β-DG [23], [24]. Together with β-DG (encoded by the same gene as α-DG, *Dag1*), it links the extracellular matrix (ECM) and cytoskeleton [24], [25] in that α-DG binds with high affinity to several extracellular matrix components, including laminin [26]–[30], agrin [31], [32], perlecan [33], [34], neurexin [35], and pikachurin [36], while β-DG interacts with cytoskeletal elements. Glycosylation mediates α-DG interaction with the extracellular matrix based on studies using monoclonal antibodies IIH6C4 and VIA4-1 that recognize glycosylated epitopes of α-DG [24], [27]. Specifically, hypoglycosylation leads to the loss of immunoreactivity to IIH6C4 (and VIA4-1) [37]–[40]. Moreover, hypoglycosylation of α-DG resulting from mutations in *POMT1*, *POMT2*, and *POMGnT1,* as well as mutations in *LARGE, FKTN, and FKRP* significantly reduces laminin binding activity [37]–[42].

LARGE is one of the largest genes in the human genome with two putative glycosyltransferase domains [43]. Large^myd^ mice bear an intragenic deletion in the *Large* gene [41], and exhibit neuronal migration defects in the brain and eye abnormalities similar to CMDs in humans [44]. LARGE interacts with the N-terminal domain of α-DG [45], and point mutations in the transferase domains abolish glycosylation activity, suggesting that LARGE functions as a glycosyltransferase [17]. Overexpression of LARGE leads to hyperglycosylated α-DG in that IIH6C4 immunoreactivity migrates at a higher apparent molecular mass on SDS-PAGE, compared to wildtype [46]. Interestingly, LARGE overexpression results in restoration of laminin binding activity in cells isolated from not only *Large^myd^* mice, but also patients with WWS, MEB, and FCMD. The ability to hyperglycosylate α-DG and “rescue” its laminin binding activity is unique to LARGE and its homolog LARGE2 [46]–[48]. These studies raise the hope of using LARGE in gene therapy for all congenital muscular dystrophies caused by defective α-DG glycosylation.

In this study, we examined whether LARGE could regulate the glycosylation of glycoproteins other than DG. Overexpression of LARGE was studied in DG-deficient neural stem cells using immunobloting with IIH6C4 and VIA4-1 antibodies in conjunction with a laminin binding assay. Our results show that LARGE glycosylates at least one glycoprotein in addition to α-DG that confers laminin binding activity.

## Materials and Methods

### Ethics statement

Protocols for animal usage were approved by the Institutional Animal Care and Use Committee of Upstate Medical University (Permit Number: 066) and adhered to National Institutes of Health guidelines. All surgery was performed under anesthesia with sodium pentobarbital. All efforts were made to minimize suffering.

### Antibodies

Antibodies were obtained as follows: Anti-α-DG antibodies IIH6C4 and VIA4-1 [27] from Millipore Corporation (Billerica, MA); Anti-β-DG (MANDAG2-7D11) from Developmental Studies Hybridoma Bank (University of Iowa, Department of Biology); Rabbit polyclonal antibodies against laminin-1 and c-Myc from Sigma-Aldrich (St. Louis, MO); Anti-β-DG (43DAG1/8D5) from Abcam (Cambridge, MA); β1 integrin blocking antibody [49] from Biolegend (San Diego, CA).

### Neural stem cell culture

To obtain brain-specific DG-deficient neural stem cells, *Dag1*-floxed mice [50] were crossed with Nestin-Cre transgenic mice [51] (both from the Jackson Laboratories, Bar Harbor, ME). Genotyping of *Dag1*-floxed allele and Nestin-Cre transgene was carried out by polymerase chain reaction (PCR). For Nestin-Cre, primers were recommended by the Jackson Laboratories. For *Dag1*-floxed allele, the wildtype primers were GGGAGAGGACTCGAACACTG and GTCTGGGGAGAAGGAAGGTC, whereas the floxed primers were TGAATGAACTGCAGGACGAG and ATACTTTCTCGGCAGGAGCA. The predicted amplicons for wildtype and floxed alleles were 243 bp, and 160 bp, respectively.

Primary neural stem cell cultures were isolated from embryonic day (E) 13.5 fetal brains (noon on the date of plug observation was considered E 0.5) of wildtype and knockout (Dag1^f/f;Nestin-Cre+^) fetuses. The neocortical wall was excised, trypsinized, and triturated. The dissociated cells were cultured as neural spheres in neural basal medium (Invitrogen, Carlsbad, CA) supplemented with B27 (minus vitamin A), 20 ng/ml FGF-2, 20 ng/ml EGF, and 2 ng/ml heparin.

To obtain clones of DG knockout neural stem cells, the neural spheres were trypsinized and triturated into individual cells. Ten ml of diluted cells (at a concentration of 6 cells/ml) were seeded in a 100 mm×15 mm fibronectin-coated tissue culture dish. Fresh FGF-2, EGF, and heparin were added once every three days. The individual colonies were picked with a pipettor and subcultured in a 12-well plate with 1 ml culture medium for a week. The cloned neural stem cells were expanded as neural spheres. Genomic DNA was then extracted from each clone and genotyped by PCR to identify *Dag1* null locus (*Dag1* locus with sequences flanked by loxP sites deleted by Cre) with the following primers: GGCCTTCCTCTTGACACTGA and GGACAGTCACTGGCTCATCA. The expected PCR fragment for the wildtype was a 217 bp amplicon. The *Dag1* null locus does not generate a fragment. *Dag1* null locus was confirmed by a pair of mutant primers CGAACACTGAGTTCATCC and CAACTGCTGCATCTCTAC [46]. The predicted ampicon for the mutant allele was 585 bp. The wildtype locus does not generate a fragment. Detection of immunoreactivity to IIH6C4 and VIA4-1, laminin overlay, and antibody blocking experiments on DG knockout cells were repeated in at least two independent clones.

RT-PCR with primers GCTCATTTCGAGTGAGCATTCC and CTAGTTTCCAGGACAGGAGA was used to determine the expression of dystroglycan mRNA [46]. These primers anneal to exon 3 and exon 4 of *Dag1* locus and produce a 561 bp amplicon when dystroglycan mRNA is expressed. Dystroglycan knockout neural stem cells were not expected to generate a fragment. RT-PCR for 18S rRNA was used as a normalization control [52]. *Dag1* null neural stem clones were further evaluated by Western blot analysis with anti-β-DG antibody and immunofluorescence staining with a β-DG antibody. Anti-β-DG immunoreactivity was undetectable in *Dagl* null clones, whereas a protein of apparent molecular mass of 43 kDa was detected in wildtype cells.

### Western blot and Laminin overlay experiments

Cultured neural spheres were pooled by centrifugation and disrupted using a Dounce homogenizer and cold lysis buffer (50 mM Tris-HCl, pH 7.4, 150 mM NaCl, 1% TritonX-100) supplemented with a protease inhibitor cocktail (Roche Diagnostics, Indianapolis, IN) then centrifuged at 16,100 *g* for 20 min at 4°C. The supernatants were collected, and 50 µl of wheat germ agglutinin (WGA)-agarose (EY Laboratories, San Mateo, CA) was added to 2 mg of total protein lysate. After incubation for 4 hrs, the WGA-gel was centrifuged and washed 3 times with the lysis buffer. Bound glycoproteins were eluted by SDS-PAGE loading dye, separated on SDS-PAGE, and electrotransferred onto polyvinylidene fluoride (PVDF) membranes.

For immunoblotting analysis with IIH6C4, MANDAG2-7D11, and anti-c-Myc antibodies, PVDF membranes were blocked with 3% BSA in TBST (50 mM Tris, pH 7.4, 150 mM NaCl, 0.05% Tween-20) for 30 min and incubated with primary antibodies in TBST and 3% BSA for two hrs. After washing with TBST, the membranes were incubated with goat anti-mouse IgG (or IgM) or rabbit IgG conjugated with horseradish peroxidase (1∶3000) for 45 min. After extensive washing with TBST, the signal was visualized with SuperSignal west pico chemiluminescent substrate (Thermo Scientific).

For immunoblotting analysis with VIA4-1, after primary antibody incubation and washing, the PVDF membrane was incubated with biotinylated goat anti-mouse IgG and washed. The membrane was then incubated with peroxidase-conjugated streptavidin, and the signal visualized as above.

For immunoprecipitation with VIA4-1 antibody, 1.5 mg of total lysate protein was used. Affinity purified VIA4-1 was mixed with the supernatant and incubated for 2 hrs at 4°C. Ten µl of protein G beads (Thermo Scientific, Rockford, IL) were added and rotated overnight. Beads were washed 3 times with washing buffer (50 mM Tris-HCl, pH 7.4, 150 mM NaCl, and 0.1% Triton X-100) at 4°C. The immunoprecipitated proteins were then eluted with 5X SDS-PAGE loading dye, boiled for 5 minutes, separated by 8% gel, and electrotransferred onto PVDF membranes.

For the laminin-overlay assay, PVDF membranes were incubated with Tris-buffered saline (TBS, 50 mM Tris, pH 7.4, 150 mM NaCl) containing 3% BSA, 1 mM CaCl_2_, and 1 mM MgCl_2_ for one hr to block nonspecific binding. The membranes were then incubated with 1.25 µg/ml laminin-1 (Invitrogen) in TBST containing 1 mM CaCl_2_ and 1 mM MgCl_2_ overnight at 4°C. After extensive washing, bound laminin was detected by standard Western blot procedures as above except that all buffers contained 1 mM CaCl_2_ and 1 mM MgCl_2_.

### Adenoviral overexpression of LARGE

An adenoviral vector for overexpression of myc-tagged human *LARGE* (Ad-LARGE) was constructed at Vector Biolabs (Philadelphia, PA). Following the coding region of LARGE, a DNA segment containing internal ribosome entry site-enhanced green fluorescent protein (IRES-eGFP) sequence was inserted to co-express eGFP as a reporter. Neural stem cells were cultured in 150 mm×20 mm dishes. The Ad-LARGE virus (20 µl of 5×10^12^ viral particles/ml) was added to the culture medium when the cells were 50%–70% confluent. Two days after infection, cells were harvested for Western blot analyses.

### Removal of N-glycans

Peptide N-glycosidase F (PNGase F) (New England Biolabs, Inc., Ipswich, MA) treatment to remove N-glycans of glycoproteins in VIA4-1 immunoprecipitates was carried out according to manufacturer's suggestions. Proteins were denatured with 1X glycoprotein denature buffer by incubating at 95°C for 10 min. After quick chilling on ice, reaction buffer containing proteinase inhibitor cocktail (Roche) was added. PNGase F (50 units) was then added to the mixture and incubated at 37°C for 16 hrs. As a control, heat inactivated PNGase F was added to the control samples and incubated likewise.

### Laminin binding on neural stem cells and semi-quantitative analysis

Neural stem cells were grown on 8-well chamber slides coated with fibronectin. Two days after infecting with the Ad-LARGE virus, Laminin-1 (Invitrogen) was added to the culture medium and incubated for 1, 6, and 12 hrs. For IIH6C4 antibody blocking experiment, the antibody was diluted 20-fold in culture medium, and cells were incubated for 2 hrs before exposure to laminin for 6 hrs. Cells were then washed 3 times with culture medium and fixed with 4% paraformaldehyde for 15 min. Bound laminin was detected by immunofluorescence staining with anti-laminin-1 antibody. Primary and secondary antibody incubations were performed as described previously [53], [54] and counterstained for 10 min with 0.10% 4′,6-diamidino-2-phenylindole (DAPI) (Sigma-Aldrich). Fluorescence was visualized with a Zeiss Axioskop upright fluorescence microscope equipped with a digital camera with 40X objective (Carl Zeiss Microimaging, Inc., Thornwood, NY).

To quantify bound laminin, digital immunofluorescence images were taken in quadruplicate at the same exposure condition such that the brightest spot was below saturation. The images were analyzed with ImageJ Software (NIH, ImageJ 1.41) such that the integrated density of immunofluorescence (sum of the pixel values in the area of interest) was measured. The integrated density of the images from cells without laminin treatment was considered 0 and used to establish the threshold for subtraction of background fluorescence in other images. For each time point, the mean and its standard error were then calculated. Cell numbers on the images were counted manually from DAPI fluorescence. To analyze individual laminin aggregates, the numbers and integrated density were determined by using the “Analyze Particles” function. To analyze filament-shaped versus dot-shaped aggregates, an aggregate with “Circularity” (4π×Area/Perimetre^2^) of 0 to 0.7 was considered a filament-shaped aggregate while an aggregate with “Circularity” of 0.7 to 1 was considered dot shaped.

## Results

### Isolation and characterization of dystroglycan knockout neural stem cells

**Figure 1:**
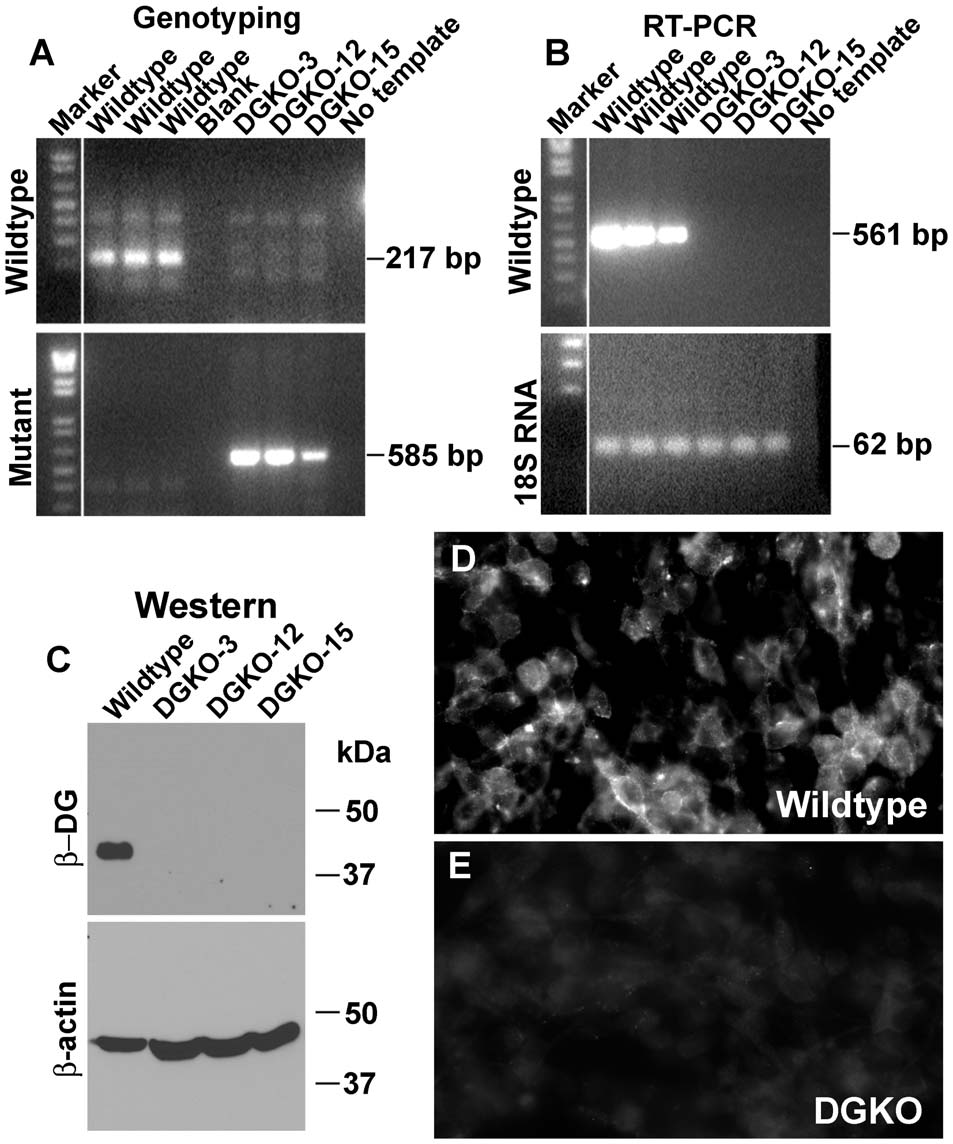
Establishment of dystroglycan-deficient neural stem cells. Primary neural stem cells isolated from brain specific knockout fetuses of dystroglycan were clonally expanded, and genotyping performed with knockout specific primers specifying intron 3. Western blot with β-DG antibody, and immunoflurescence staining with β-DG antibody were carried out to confirm successful knockout in clones. (A) Genotyping. (B) RT-PCR. (C) Western blot with β-DG antibody. (D and E) Anti-β-DG immunofluorescence staining of wildtype and knockout neural stem cells respectively. Abbreviations: DG, dystroglycan; DGKO, dystroglycan knockout. Scale bar in E: 50 µm.

To obtain brain-specific knockout of α-DG, *Dag1*-floxed mice were crossed with Nestin-Cre transgenic mice to produce Dag1^f/f;Nestin-Cre+^. These mice showed similar central nervous system phenotypes as GFAP-Cre-mediated knockout of DG [50]. Neural specific deletion of DG would not affect DG expression in non-neural cells such as blood vessels. Therefore, to obtain DG-null cells for biochemical analysis, we isolated neural stem cells from Dag1^f/f;Nestin-Cre+^ fetuses at E13.5, and verified that these cells were immunoreactive to the neural stem cell marker RC2. To ensure that the cells were DG deficient, cell cultures were clonally expanded, and ten clones were isolated and genotyped by PCR. As expected, these expressed a 585 bp ampicon specifying the mutant clones (DGKO-3, DGKO-12, and DGKO-15 as examples), while the wildtype neural stem cells expressed the 217 bp amplicon (**Figure 1A**). Loss of dystroglycan gene expression was confirmed by RT-PCR (**Figure 1B**). Consistent with this, β-DG immunoreactivity was undetectable in the knockout clones, while the wildtype clones expressed a 43 kDa protein on Western blot (Figure 1C) and immunocytochemistry (**Figures 1D, E**). Moreover, cells rendered null for α- and β-DG did not show noticeable changes in cellular morphology or growth rate as compared with the wildtype cells. Altogether, these results indicate that the putative knockout clones were dystroglycan nulls showing no detectable expression of DG mRNA or protein.

### Overexpression of LARGE in DG-deficient neural stem cells caused hyperglycosylation of non-α-DG targets

**Figure 2:**
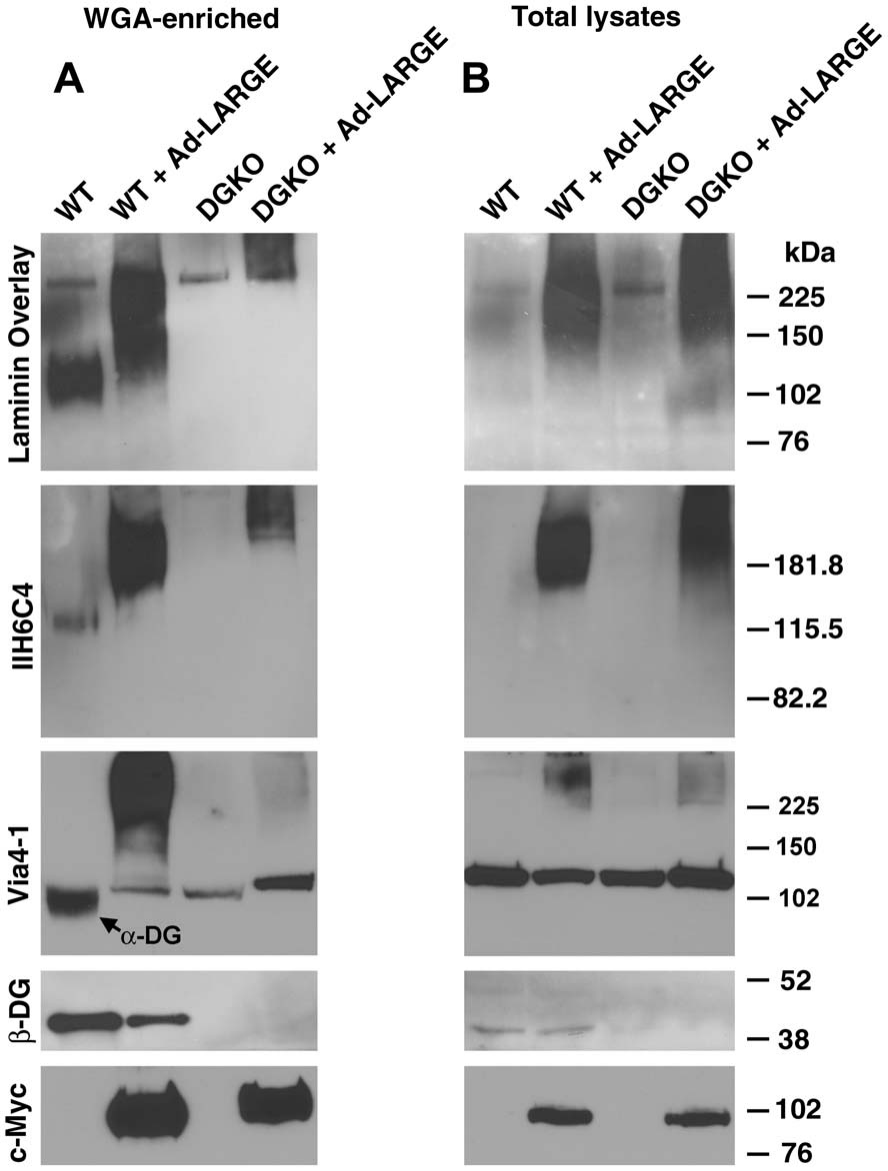
LARGE can hyperglycosylate glycoprotein(s) in DG-deficient neural stem cells that bind to laminin. Clonally expanded Dag1 null neural stem cells were infected with the Ad-LARGE virus. WGA-enriched glycoproteins were then isolated from total cell lysates, separated on SDS-PAGE, and blotted onto PVDF membranes. Laminin overlay, immunoblot with IIH6C4, VIA4-1, and anti-β-DG were then carried out. Note that IIH6C4 and VIA4-1 immunoreactivity and laminin binding was not detectable in DG-deficient cells but was detectable in DG-deficient neural stem cells with LARGE overexpression. The 225 kDa band in laminin overlay assays were from endogenously expressed laminin (not the exogenous laminin). Abbreviations: DG, dystroglycan; DGKO, DG knockout; kDa, kilo Dalton; WGA, wheat germ agglutinin; WT, wildtype.

To examine whether there are non-α-DG targets for LARGE-mediated glycosylation, we infected DG-deficient neural stem cell clones with the Ad-LARGE virus. Two days later, WGA-enriched glycoproteins were isolated from cell lysates and analyzed with IIH6C4 antibody, laminin overlay, and anti-β-DG Western blot. In control neural stem cells that were Cre-negative (considered as wildtype, WT), IIH6C4 immunoreactivity was detected at about 120 kDa (**Figure 2A**). IIH6C4 immunoreactive α-DG bound to laminin in the laminin overlay assay. Overexpression of LARGE in the controls (WT + Ad-LARGE) increased the amount of IIH6C4 immunoreactivity, and the apparent molecular mass of immunoreactive proteins. IIH6C4 immunoreactive species in LARGE-overexpressing wildtype cells migrated as a smear. Laminin overlay indicated laminin binding activity was increased by LARGE overexpression in wildtype cultures. As expected, β-DG was detected in the WGA-enriched glycoproteins from wildtype cells, and wildtype cells overexpressing LARGE.

In DG-deficient neural stem cells (DGKO), IIH6C4 immunoreactivity and laminin binding activity were not detected at ∼120 kDa, indicating the absence of α-DG (**Figure 2A**). Also as expected, β-DG immunoreactivity was undetectable, indicating that the cells were deficient in total DG. These results were expected because IIH6C4 is thought to recognize a carbohydrate epitope on α-DG. Unexpectedly, strong IIH6C4 immunoreactivity was detected in the DG-deficient neural stem cells overexpressing LARGE (DGKO + Ad-LARGE). The IIH6C4 immunoreactive species did not belong to recombinant LARGE since the Myc-tagged LARGE migrated at ∼100 kDa (**Figure 2**, both panels). In addition, LARGE overexpression in DG-deficient cells restored laminin binding. In DG-deficient cells overexpressing LARGE, the IIH6C4 immunoreactive and laminin-binding protein species migrated at a higher molecular weight than wildtype cells overexpressing LARGE (compare lanes WT + Ad-LARGE with DGKO + Ad-LARGE). The laminin overlay experiment also detected a 225 kDa protein of endogenous laminin expressed by DG-deficient cells as well as wildtype cells. As a control, β-DG was not detected in DG-deficient cells with or without LARGE overexpression. Anti-c-Myc specifically detected Myc-tagged LARGE in cells treated with the Ad-LARGE virus. There was no apparent difference in expression of LARGE in wildtype and knockout cells. These results indicate that there are non-α-DG protein species hyperglycosylated by LARGE in WGA-enriched glycoprotein fractions.

Similar results were obtained using the VIA4-1 antibody. VIA4-1 immunoreactivity was observed at 120 kDa in lysates from wildtype cells (arrow in **Figure 2A**). The use of biotin-avidin amplification technique in VIA4-1 immunoblot produced a nonspecific band migrating at 125 kDa. LARGE overexpression caused a dramatic increase in VIA4-1 immunoreactivity similar to IIH6C4 immunoreactivity (**Figure 2A**). In dystroglycan-deficient neural stem cells, VIA4-1 immunoreactivity was not detectable. However, LARGE overexpression produced VIA4-1 immunoreactive species at high molecular weight range as well (**Figure 2A**).

**Figure 3:**
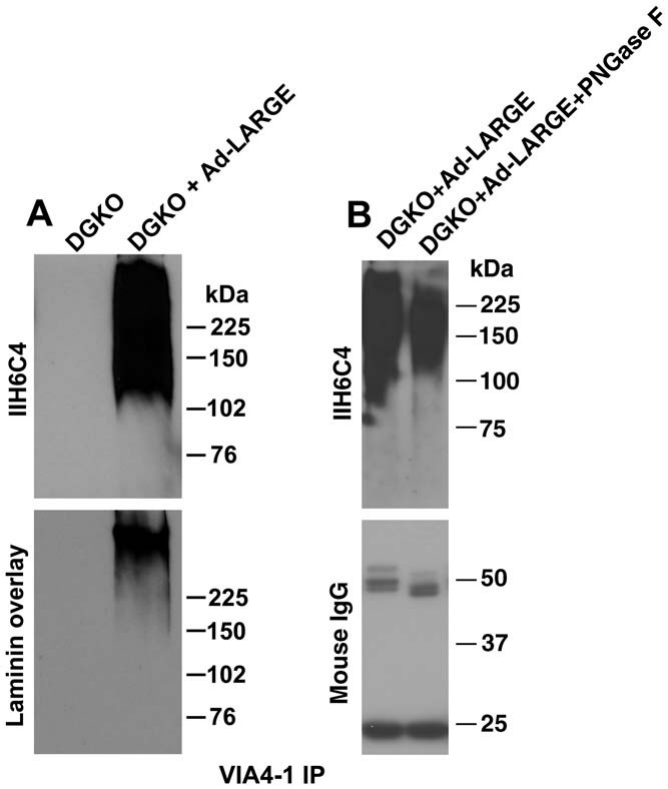
Hyperglycosylated proteins in DG deficient neural stem cells were also recognized by VIA4-1 and some were sensitive to peptide N-glycosidase F. Lysates of DG-deficient neural stem cells with or without LARGE overexpression were immunoprecipitated with VIA4-1 antibody. (A) The immunoprecipitates were analyzed by immunoblotting with IIH6C4. (B) The VIA4-1 immunoprecipitates were treated with PNGase F and analyzed by immunoblotting with IIH6C4. Abbreviation: DGKO, dystroglycn knockout; IP, immunoprecipitation.

In addition, we used VIA4-1 antibody to immunoprecipitate proteins from lysates of LARGE-overexpressing DG-deficient cells. The immunoprecipitate from LARGE overexpressing DG-deficient cells showed strong immunoreactivity to IIH6C4 and bound to laminin in the laminin overlay assay (**Figure 3A**). IIH6C4 antibody did not work in the immunoprecipitation assay. These results indicate that a substrate (s) other than α-DG is hyperglycosylated when LARGE is overexpressed, which is recognized by IIH6C4 and VIA4-1 and binds to laminin.

In wildtype cells, IIH6C4 immunoreactivity and laminin binding could not be detected from total lysate (**Figure 2B**). However, strong IIH6C4 immunoreactivity and laminin binding were observed in total lysate of wildtype cells with overexpression of LARGE (WT + Ad-LARGE). Similarly, IIH6C4 and VIA4-1 immunoreactivity and laminin binding were also seen in total lysates of DG-deficient cells after overexpression of LARGE (DGKO + Ad-LARGE). As expected, β-DG was not detected in total lysates of DG-deficient cells with or without LARGE overexpression.

### Peptide N-glycosidase F treatment only slightly reduced IIH6C4 immunoreactivity

A significant amount of IIH6C4 immunoreactivity in LARGE overexpressing Chinese hamster ovary cells were N-linked because they were sensitive to PNGase F treatment [16]. To determine whether LARGE overexpression modified N-glycans of non-α-DG glycoproteins, we treated VIA4-1 immunoprecipitated proteins from LARGE overexpressing DG-deficient cells with PNGase F and immunoblotted with IIH6C4 (**Figure 3B**). There was a modest reduction in IIH6C4 immunoreactivity upon PNGase F treatment. When IIH6C4 immunoreactivity from 3 independent experiments was quantified by densitometric analysis, it revealed that PNGase F reduced IIH6C4 immunoreactivity by 16.3% (95% confidence interval: 24.9% to 2.9%). These results indicate that LARGE can modify N-glycans on some non-α-DG glycoproteins.

### LARGE-hyperglycosylated non-α-DG glycoprotein(s) were located on the plasma membrane

**Figure 4:**
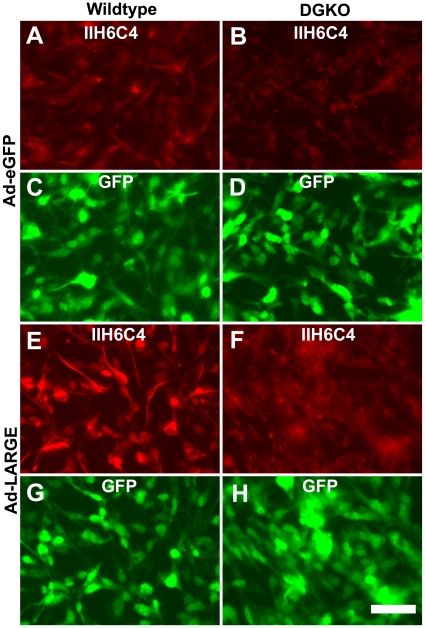
Overexpression of LARGE increased IIH6C4 immunoreactivity on DG-deficient neural stem cells. Neural stem cells were cultured on fibronectin-coated chamber slides and infected with the Ad-EGFP (A–D) and Ad-LARGE viruses (E–H). Two days later, cells were fixed and immunostained with IIH6C4 antibody. (A, C, E, and G) Wildtype neural stem cells. (B, D, F, and H) DG-deficient neural stem cells. Note that LARGE overexpression increased IIH6C4 immunoreactivity in both the wildtype and DG-deficient neural stem cells (compare E and F to A and B). Abbreviations: DGKO, DG knockout; WT, wildtype. Scale bar in H: 50 µm.

**Figure 5:**
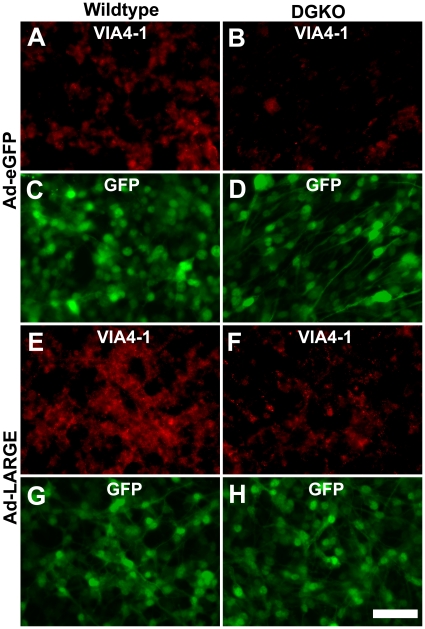
Overexpression of LARGE increased VIA4-1 immunoreactivity on DG-deficient neural stem cells. Neural stem cells were cultured on fibronectin-coated chamber slides and infected with the Ad-EGFP (A–D) and Ad-LARGE viruses (E–H). Two days later, cells were fixed and immunostained with VIA4-1 antibody. (A, C, E, and G) Wildtype neural stem cells. (B, D, F, and H) DG-deficient neural stem cells. Note that LARGE overexpression increased VIA4-1 immunoreactivity in both the wildtype and DG-deficient neural stem cells (compare E and F to A and B). Abbreviations: DGKO, DG knockout; WT, wildtype. Scale bar in H: 50 µm.

To determine whether LARGE overexpression increased IIH6C4 immunoreactivity on the cells, we carried out immunofluorescence staining of neural stem cells that were infected with the Ad-LARGE virus. In wildtype cells, IIH6C4 immunofluorescence was readily observed (**Figure 4A**). Overexpression of LARGE increased the intensities of IIH6C4 immunofluorescence (**Figure 4E**). In DG-deficient cells, IIH6C4 immunofluorescence was barely detected (**Figure 4B**). However, overexpression of LARGE increased IIH6C4 immunofluorescence in these cells (**Figure 4F**). When cells were examined by indirect immunofluorescence with the VIA4-1 antibody, similar results were obtained (**Figure 5**) in that LARGE overexpression increased immunofluorescence intensities both in the wildtype cells and DG-deficient cells (compare **Figure 5E** with **A** and **F** with **B**). The staining pattern with VIA4-1, however, was slightly different from that of IIH6C4. VIA4-1 immunofluorescence showed a more punctate pattern with cellular processes rarely stained.

### LARGE overexpression in DG-deficient cells promoted laminin binding at the cell surface

**Figure 6:**
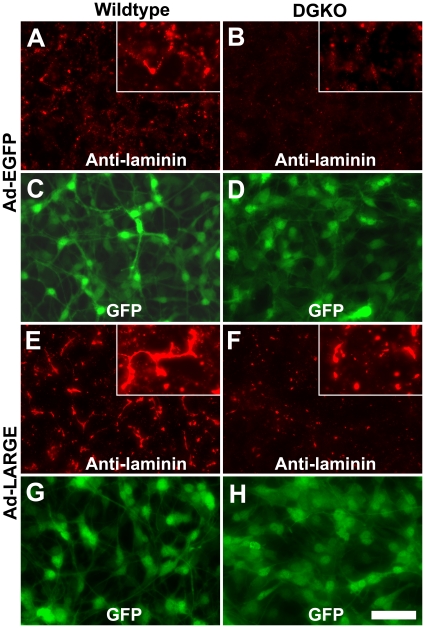
Overexpression of LARGE in DG-deficient neural stem cells promoted laminin binding at the cell surface. Neural stem cells were cultured on fibronectin-coated chamber slides and infected with Ad-EGFP (A–D) and Ad-LARGE viruses (E–H). Two days after infection, laminin was added to the medium. The cells were washed and fixed 12 hrs later and immunostained with an antibody against laminin (red fluorescence, A, B, E, and F). (A, C, E, and G) Wildtype neural stem cells. (B, D, F, and H) DG-deficient neural stem cells. Abbreviations: DGKO, DG knockout; WT, wildtype. Scale bar in H: 50 µm (25 µm for inserts in A, B, E, and F).

**Figure 7:**
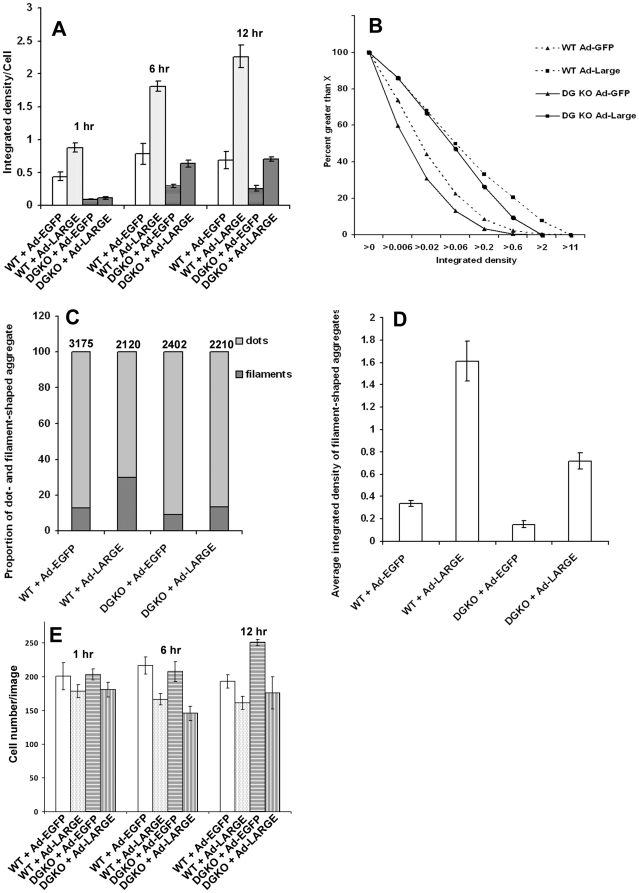
Quantification of laminin binding on neural stem cells. (A) Comparison of overall laminin immunofluorescence intensities for wildtype and DG-deficient neural stem cells with or without LARGE overexpression after incubating with laminin for 1, 6, and 12 hrs. (B) Distribution of aggregate fluorescence intensities. Y-axis shows the percentage of laminin aggregates with fluorescence intensities greater than those shown on the X-axis. (C) Quantification of filamentous and dot–shaped laminin aggregates. (D) Average fluorescence intensities of filamentous aggregates in wildtype and DG-deficient cells with or without overexpression of LARGE. (E) Number of cells in the images analyzed for (A). Images from the 12 hour data point were used for (B, C, and D). Abbreviations: DGKO, DG knockout; WT, wildtype.

Functionally glycosylated α-DG is not only recognized by IIH6C4 antibody but also binds to extracellular matrix molecules such as laminin. To determine whether non-α-DG glycoproteins hyperglycosylated by LARGE overexpression promote laminin assembly at the cell surface, we added laminin to the cell culture medium and assayed for bound laminin by immunofluorescence staining. In the absence of LARGE overexpression, laminin binding was detected on wildtype neural stem cells (**Figure 6A**). LARGE overexpression augmented the amount of laminin binding as revealed by overall increase in anti-laminin immunofluorescence (**Figure 6E**). To quantify the effect of LARGE overexpression, we measured fluorescence intensities and found that overexpression of LARGE in wildtype cells dramatically increased the overall immunofluorescence intensities of bound laminin after 1 hr, 6 hrs, and 12 hrs of laminin incubation (**Figure 7A** **,** p<0.001, Student's t-test).

DG-deficient neural stem cells showed detectable but markedly reduced amounts of bound laminin when compared to the wildtype indicated by much weaker immunofluorescence (compare **Figure 6B** with **A**). The residual laminin binding was likely due to the presence of other laminin receptors such as integrins. Overexpression of LARGE in DG-deficient neural stem cells also increased binding of added laminin (compare **Figure 6F** with **B**). When fluorescence intensities were measured, the laminin immunofluorescence in DG-deficient cells was dramatically decreased from the wildtype at all three time points. However, LARGE overexpression markedly increased laminin immunofluorescence intensities after 6 hrs and 12 hrs indicating increased laminin binding (**Figure 7A**, p<0.01, Student's t-test). Although laminin binding in DG-deficient cells with LARGE overexpression could reach levels comparable to the wildtype neural stem cells (**Figure 7A**, 6 hrs and 12 hrs), it did not reach the level of wildtype cells overexpressing LARGE at any of the time points.

It appeared that LARGE overexpression increased the total amount of bound laminin by boosting the amount of laminin per aggregates as indicated by their larger sizes and stronger fluorescence intensities. To quantify this effect, we first measured the fluorescence intensities of the aggregates after 12 hrs of incubation with laminin. The aggregates were grouped according to their integrated fluorescence intensity (X-axis) in **Figure 7B**. The percentage of aggregates with fluorescence intensity greater than intensity indicated on the X-axis is shown on the Y-axis. DG-deficient cells exhibited a leftward shift of the curve from the wildtype, suggesting decreased average fluorescence intensity from the wildtype. Overexpression of LARGE in both the wildtype and DG-deficient cells shifted the curve to the right, suggesting increased average fluorescence intensity compared to cells without LARGE overexpression. We also measured the areas occupied by the laminin aggregates with and without LARGE overexpression which resulted in area measurements similar to fluorescence intensity measurement. Overexpression of LARGE increased the average area occupied by the laminin aggregates in both the wildtype cells and DG-deficient cells (data not shown).

Bound laminin aggregated into various sizes with two frequent physical appearances: dot-shaped and filament-shaped aggregates. Filament-shaped laminin aggregates were generally much larger than dots with brighter overall fluorescence. In the wildtype, 12.63% of aggregates were filamentous (**Figure 7C**). Overexpression of LARGE increased the filamentous aggregates to 29.91% (also compare **Figure 6E** with **A**, Chi-square analysis, X^2^ = 222.27, p<0.001). In DG-deficient cells, LARGE overexpression increased filamentous aggregates from 9.08% to 13.21% (also compare **Figure 6F** with **B**, Chi-square analysis, X^2^ = 21.88, p<0.001). Thus, similar to the wildtype, bound laminin with filamentous morphologies were more frequently observed in DG-deficient cells with LARGE overexpression than without LARGE overexpression. In addition, the fluorescence intensities of filamentous aggregates were markedly increased after overexpression of LARGE in both the wildtype and DG knockout cells (**Figure 7D**). Taken together, these data indicate that overexpression of LARGE in DG-deficient neural stem cells increases laminin binding at the cell surface, suggesting that non-α-DG targets of LARGE-mediated glycosylation also mediate laminin binding upon overexpression of LARGE.

**Figure 8:**
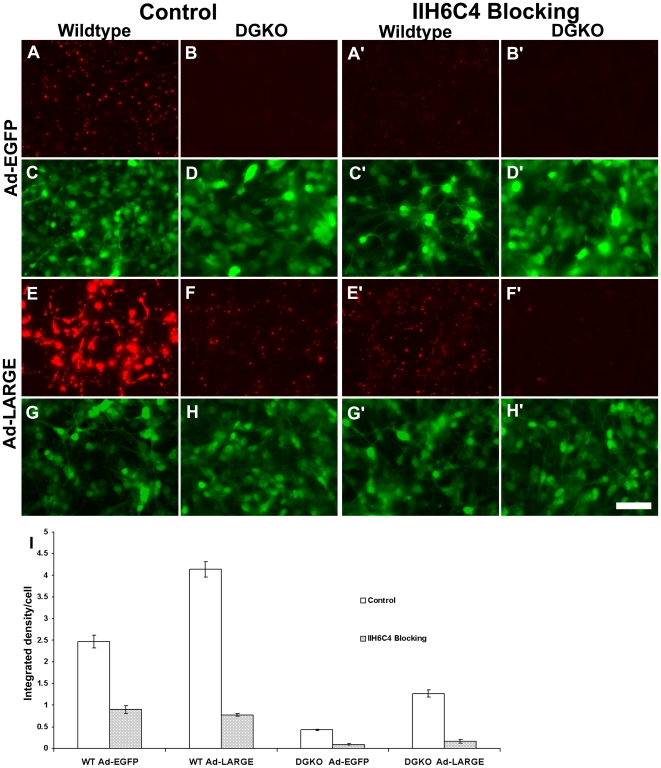
Laminin binding by non-α-DG glycoproteins in LARGE overexpressing DG-deficient cells was blocked by IIH6C4. IIH6C4 antibody was added to the culture medium of neural stem cells, laminin was added, and bound laminin was detected by immunofluorescence staining. (A, C, E, and G) Wildtype neural stem cells. (B, D, F, and H) DG-deficient neural stem cells. (A', C', E', and G') Wildtype neural stem cells treated with IIH6C4. (B', D', F', and H') DG-deficient neural stem cells treated with IIH6C4. (I) Quantification of bound laminin. Scale bar in H': 50 µm.

Laminin binding by α-DG is blocked by the IIH6C4 antibody [55]. To determine whether laminin binding activity by LARGE-modified non-α-DG is sensitive to IIH6C4, a IIH6C4 blocking experiment was performed. As expected, IIH6C4 pretreatment inhibited laminin binding to wildtype neural stem cells with or without LARGE overexpression (compare **Figure 8**A' and E' with A and E). While LARGE overexpression increased laminin binding in dystroglycan-deficient cells, IIH6C4 pretreatment significantly reduced laminin binding (compare **Figure 8**F' with F). As a control, an antibody that has been shown to block β1-integrin function did not affect laminin binding in wildtype and dystroglycan-deficient cells with or without LARGE overexpression (data not shown). Quantitative analysis of bound laminin indicated that IIH6C4 inhibited laminin binding in LARGE-overexpressing dystroglycan-deficient cells as well (**Figure 8I**). IIH6C4 treatment reduced laminin binding in all cells (p<0.01 for all comparisons, Student t test). These results indicate that laminin binding activity of non-α-DG proteins upon LARGE overexpression is not only recognized by IIH6C4 but also can be blocked by it.

## Discussion

To date, DG is the only known substrate of the six glycosyltransferases (or presumed glycosyltransferases) (POMT1, POMT2, POMGnT1, LARGE, Fukutin, and FKRP) mutated in CMDs. To determine if the presumed glycosyltransferase LARGE recognizes other substrates, it was overexpressed in DG-deficient neural stem cells. Western blot analysis revealed the presence of IIH6C4 and VIA4-1 immunoreactive species in WGA-enriched glycoproteins and in total lysates, supporting glycosylation of a non-α-DG protein in neural stem cells overexpressing LARGE. Moreover, laminin overlay analysis demonstrated that this substrate(s) was capable of binding to laminin, and LARGE overexpression promoted cell surface binding of laminin in DG-deficient cells. Altogether, these results indicate that LARGE can regulate the glycosylation of a glycoprotein(s) in addition to α-DG.

### IIH6C4 and VIA4-1 epitopes are transferred to non-α-DG upon LARGE overexpression

Immunoreactivity to two antibodies, IIH6C4 and VIA4-1, has been widely used to evaluate functional glycosylation of α-DG on unidentified carbohydrate epitopes [24], [27]. Mutations in *POMT1*, *POMT2*, and *POMGnT1, LARGE, FKTN, and FKRP* lead to hypoglycosylation of α-DG and markedly reduce ligand binding activity [37]–[42], [56], [57]. Interestingly, LARGE overexpression produced IIH6C4 immunoreactive protein species that bind laminin in cells isolated from both Large^myd^ mice and patients with WWS, MEB, and FCMD [46]. It has been assumed that α-DG is the only hyperglycosylated protein species that is produced by LARGE overexpression [16], [46]–[48], [58]. Our results show that overexpressing LARGE produces glycoproteins recognized by IIH6C4 and VIA4-1 in DG-deficient cells. It is not known why they were more immunoreactive to IIH6C4 than VIA4-1 (Figure 2). It is possible that LARGE-mediated modifications on α-DG and proteins other than α-DG are not identical. Nevertheless, these results indicate that non-α-DG species become acceptors of LARGE-mediated glycosylation that are recognized by IIH6C4 and VIA4-1.

### LARGE as a glycosyltransferase

Several lines of evidence suggest that LARGE is a glycosyltransferase. First, it has two glycosyltransferase domains, one similar to β-1,3-N-acetylglucosaminyltransferase and the other similar to UDP-glucose:glycoprotein glucosyltransferase (UGGT) [16], [17], [41]. Second, site-directed mutation of conserved DXD motifs within the transferase domains abolishes LARGE's ability to generate IIH6C4 immunoreactivity by mislocalizing the mutant protein to the endoplasmic reticulum [48] or by inactivating the putative enzymatic domain [17]. Third, O-linked mannose can have four different branches: (1) N-acetylglucosamine linked through β1,2 linkage catalyzed by POMGnT1 [4], (2) N-acetylglucosamine linked through β1,6 linkages catalyzed by β1,6-N-acetylglucosaminyltransferase IX [59], [60], (3) N-acetylglucosamine linked through β-1,4, and (4) an unidentified phosphoryl glycan linked to carbon-6 [18]. The extension of the phosphoryl glycan branch requires the activity of LARGE [18]. Fourth, overexpression of LARGE in CHO cells and some cancer cells hyperglycosylate α-DG [16], [17], [58].

Prior to this work, the only known target of LARGE was α-DG and three types of glycosylations were observed: N-linked, O-linked mannosyl, and mucin type O-linked N-acetylgalactosamine glycans. The present study shows that LARGE can modify non-α-DG glycoproteins, and the modified non-α-DG species are recognized by IIH6C4 and VIA4-1. Since previous studies have generally used IIH6C4 and VIA4-1 immunoreactivity as evidence of glycosylated α-DG after LARGE overexpression, it is likely that IIH6C4-recognized species are heterogeneous, including non-α-DG as well as α-DG. Future studies are needed to determine whether LARGE hyperglycosylates one or more proteins other than α-DG in other cells and if it mediates the same glycosylation on other proteins as it does on α-DG.

In summary, this study shows that hyperglycosylation and restoration of laminin binding by LARGE overexpression also occurs in DG-deficient cells, indicating that proteins in addition to α-DG are hyperglycosylated and capable of laminin binding. It is not known whether these other targets represent one protein or several proteins. Since diminished cell-extracellular matrix interactions mediated by α-DG-laminin interaction underlie many phenotypes found in congenital muscular dystrophies, restoring cell-laminin interactions in congenital muscular dystrophies may provide therapeutic benefit. Furthermore, recent evidence suggests that silencing LARGE causes loss of laminin-α-DG binding in epithelium-derived cancers [61]. LARGE forms a complex with β3-N-acetylglucosaminyltransferase 1 (β3GnT1) and its glycosylation has tumor suppression activity [58]. Identifying the non-α-DG glycoprotein(s) glycosylated by LARGE may provide novel molecular targets for therapy.
